# Obesity-induced metabolic imbalance allosterically modulates CtBP2 to inhibit PPAR-alpha transcriptional activity

**DOI:** 10.1016/j.jbc.2023.104890

**Published:** 2023-06-05

**Authors:** Kenji Saito, Motohiro Sekiya, Kenta Kainoh, Ryunosuke Yoshino, Akio Hayashi, Song-Iee Han, Masaya Araki, Hiroshi Ohno, Yoshinori Takeuchi, Tomomi Tsuyuzaki, Daichi Yamazaki, Chen Wanpei, Lisa Hada, Sho Watanabe, Putu Indah Paramita Adi Putri, Yuki Murayama, Yoko Sugano, Yoshinori Osaki, Hitoshi Iwasaki, Naoya Yahagi, Hiroaki Suzuki, Takafumi Miyamoto, Takashi Matsuzaka, Hitoshi Shimano

**Affiliations:** 1 Department of Endocrinology and Metabolism, Institute of Medicine, University of Tsukuba, Tsukuba, Ibaraki, Japan; 2 Transborder Medical Research Center, University of Tsukuba, Tsukuba, Ibaraki, Japan

**Keywords:** CtBP2, PPARα, malonyl-CoA, metabolite sensor, fatty acid oxidation

## Abstract

Maintenance of metabolic homeostasis is secured by metabolite-sensing systems, which can be overwhelmed by constant macronutrient surplus in obesity. Not only the uptake processes but also the consumption of energy substrates determine the cellular metabolic burden. We herein describe a novel transcriptional system in this context comprised of peroxisome proliferator-activated receptor alpha (PPARα), a master regulator for fatty acid oxidation, and C-terminal binding protein 2 (CtBP2), a metabolite-sensing transcriptional corepressor. CtBP2 interacts with PPARα to repress its activity, and the interaction is enhanced upon binding to malonyl-CoA, a metabolic intermediate increased in tissues in obesity and reported to suppress fatty acid oxidation through inhibition of carnitine palmitoyltransferase 1. In line with our preceding observations that CtBP2 adopts a monomeric configuration upon binding to acyl-CoAs, we determined that mutations in CtBP2 that shift the conformational equilibrium toward monomers increase the interaction between CtBP2 and PPARα. In contrast, metabolic manipulations that reduce malonyl-CoA decreased the formation of the CtBP2–PPARα complex. Consistent with these *in vitro* findings, we found that the CtBP2–PPARα interaction is accelerated in obese livers while genetic deletion of CtBP2 in the liver causes derepression of PPARα target genes. These findings support our model where CtBP2 exists primarily as a monomer in the metabolic milieu of obesity to repress PPARα, representing a liability in metabolic diseases that can be exploited to develop therapeutic approaches.

Persistent excessive caloric intake causes spillover of fatty acids from either adipose storage or dietary intake that exerts detrimental metabolic effects (1, 2). Upon cellular uptake, fatty acids are converted into fatty acyl-CoAs, which are further partitioned into multiple metabolic processes such as lipid droplet formation to limit their intrinsic toxicity (3). Since nonadipose tissues such as liver, pancreatic β-cells, and skeletal muscle have a limited capacity for storage of lipids, excessive fatty acid influx leads to the accumulation of fatty acids as well as their CoA derivatives, defining a major metabolic liability (4, 5, 6). While storage of lipids in lipid droplets is one of the cell-intrinsic mechanisms to protect cells against the toxicity of lipids (7), cells have also evolved catabolic systems, such as lipid oxidation, to limit the lipid burden (8).

Peroxisome proliferator-activated receptor alpha (PPARα), a member of the PPAR transcription factor family, is a master regulator of fatty acid oxidation in this context (9). Of note, PPARα can be activated by synthetic and endogenous ligands including fatty acids, suggesting the key role of PPARα in the homeostatic maintenance of fatty acid metabolism (10). Since the CoA derivatives of fatty acids, which can be similarly accommodated in the ligand-binding pocket, may negatively influence the PPARα activity (11, 12), the thiol esterification of fatty acids may diminish their PPARα-activating property. Intriguingly, one of the short-chain acyl-CoA derivatives, malonyl-CoA competitively inhibits carnitine palmitoyltransferase 1 (CPT1) activity, contributing to the negative regulation of fatty acid oxidation by preventing fatty acid entry into mitochondria (13). The rate-limiting enzyme of malonyl-CoA production is acetyl-CoA carboxylase (ACC), which undergoes inhibitory phosphorylation by AMP-activated protein kinase (AMPK), and this pathway is one of the prime targets of the antidiabetic drug metformin (14).

While PPARα favorably binds to fatty acids, we have recently demonstrated that C-terminal binding protein 2 (CtBP2), a transcriptional corepressor, interacts with fatty acyl-CoAs (15). CtBP2 also has a structural pocket called a Rossmann fold which accommodates NADH/NAD^+^ with a preferential binding affinity for NADH (16, 17, 18) and confers redox-sensing capability to CtBP2 (19). Upon binding to NADH, CtBP2 adopts a dimeric configuration. Fatty acyl-CoAs bind to CtBP2 with the CoA moiety in the Rossmann fold competing with NADH, and the acyl-chain moiety at the dimerization interface physically blocks dimerization (15). This dual specificity can be explained by the (di)nucleotide-binding property of the Rossmann fold pocket in CtBP2 (20) that preferentially binds to the adenosine structure shared by the CoA moiety of fatty acyl-CoAs and nucleotide moiety of NADH. CtBP2 adopts a monomeric configuration in obese liver in response to an increase in fatty acyl-CoA content, resulting in the liberation of Forkhead box O1 (FoxO1) (21) and sterol regulatory element-binding protein 1 (SREBP1) (22) to concurrently activate hepatic gluconeogenesis and lipogenesis, a hallmark of obesity. Conversely, activation of CtBP2 in obese liver ameliorates diabetes and hepatic steatosis (15).

We herein demonstrate that monomeric CtBP2, the predominant form in the metabolic milieu associated with obesity, represses the transcriptional activity of PPARα. The CtBP2–PPARα interaction may provide a basis to better understand the pathogenesis of obesity for the development of novel therapeutic approaches.

## Results

### CtBP2 forms a repressive transcriptional complex with PPARα

**Figure 1:**
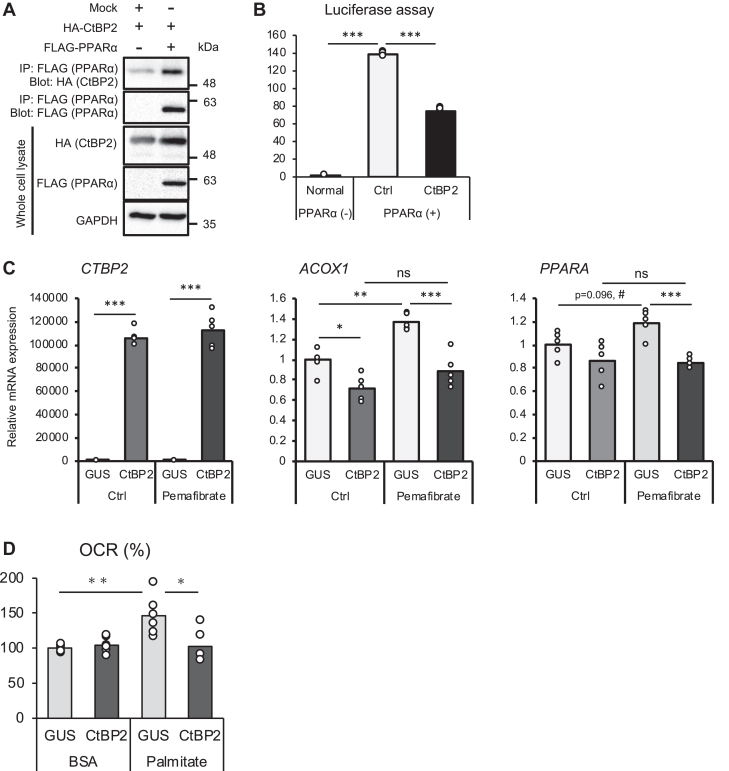
**CtBP2 forms a repressive transcriptional complex with PPARα.***A*, CtBP2 interacts with PPARα. HEK293 cells were transfected with either a control vector or FLAG-PPARα along with HA-CtBP2 plasmids. The CtBP2/PPARα transcriptional complex was analyzed by FLAG-tag co-immunoprecipitation. *B*, exogenous expression of CtBP2 reduces PPARα-mediated PPRE reporter activation in HEK293 cells (n = 4). *C*, HepG2 hepatoma cells were transduced with adenoviruses expressing control protein GUS or CtBP2 in the absence (control, Ctrl) or presence of 10 μM pemafibrate (n = 5 for each group). The expression levels of key genes were analyzed. *D*, HepG2 hepatoma cells were transduced with adenoviruses expressing control protein GUS or CtBP2, and oxygen consumption rate (OCR) induced by control BSA or BSA-conjugated palmitate (200 μM) was measured. Data are expressed as the mean ± SEM. ∗, ∗∗, and ∗∗∗ denote *p* < 0.05, *p* < 0.01, and *p* < 0.001 evaluated by one-way ANOVA followed by Tukey's multiple comparisons test. # denotes *p* < 0.05 evaluated by unpaired two-tailed Student’s *t* test. ns denotes nonstatistical significance. BSA, bovine serum albumin; CtBP, C-terminal binding protein; GUS, glucuronidase; HA, hemagglutinin; PPARα, peroxisome proliferator-activated receptor alpha; PPRE, peroxisome proliferator response element.

Based on our previous finding that CtBP2 adopts a monomeric configuration upon binding to fatty acyl-CoAs and derepresses SREBP1-mediated fatty acid biosynthesis, we hypothesized that CtBP2 may have a broad influence on fatty acid metabolism. As a first step, we examined a potential interaction with PPARα, a master regulator for fatty acid oxidation. Indeed, we were able to observe an interaction between CtBP2 and PPARα in HEK293 cells (Fig. 1*A*). Based on this finding, we surveyed the primary amino acid sequences of PPARα from several different species to examine whether they have the putative CtBP-binding site(s), Pro-x-Asp-Leu motif (23). The amino acid sequences of PPARα lacked this CtBP-binding motif, indicating an indirect interaction. While CtBP2 has been widely accepted to be a transcriptional corepressor, CtBP2 can also serve as a transcriptional coactivator in some specific cases (24, 25). Therefore, we next examined how CtBP2 modulates the PPARα transcriptional activity through this interaction. The peroxisome proliferator response element (PPRE)-driven reporter was activated by the ectopic expression of PPARα but was reduced by the expression of CtBP2 (53%), suggesting a repressive role of CtBP2 (Fig. 1*B*). We also examined the effects of CtBP2 on other PPAR isoforms and found that PPARγ may also be repressed by CtBP2. Since PPARδ did not sufficiently activate our luciferase reporter, we were not able to reach a meaningful conclusion regarding the effect on this isoform (Fig. S1*A*). In line with these findings, overexpression of CtBP2 in HepG2 cells, a human hepatoma cell line, reduced the expression levels of PPARα target genes at baseline compared to the overexpression of a control protein, glucuronidase (GUS), albeit to a moderate extent (Fig. 1*C*, 30% and 15% for acyl-CoA oxidase 1 [ACOX1] and PPARA, respectively). Pharmacological activation of PPARα with a synthetic agonist pemafibrate increased the expression levels of these genes that were blunted by CtBP2 overexpression (35% and 30% for ACOX1 and PPARA, respectively). We also examined GW7647, another widely employed PPARα agonist, where the repressive activity of CtBP2 on PPARα was reproducible (Fig. S1*B*). We further examined whether modulation of this transcriptional system exerts a functional influence on fatty acid oxidation by measuring oxygen consumption rate (OCR). As expected, CtBP2 overexpression suppressed palmitate-induced fatty acid oxidation in HepG2 cells (Fig. 1*D*).

### Preferential binding of monomeric CtBP2 to PPARα

**Figure 2:**
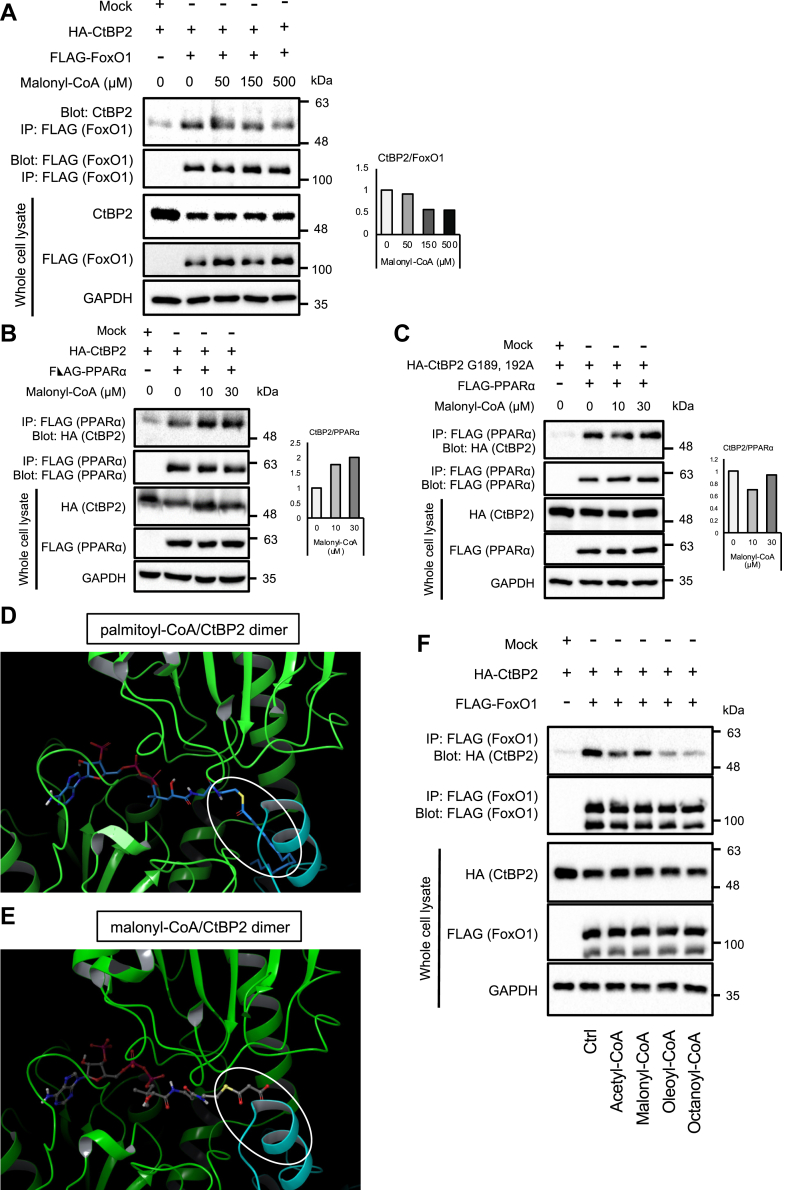
**Malonyl-CoA promotes the interaction between CtBP2 and PPARα by targeting the Rossmann fold pocket in CtBP2.***A*, increasing concentrations of malonyl-CoA were added to the cell lysates from HEK293 cells expressing WT CtBP2 along with FLAG-FoxO1. *B* and *C*, increasing concentrations of malonyl-CoA were added to the cell lysates from HEK293 cells expressing either WT CtBP2 (*B*) or Rossmann fold mutant CtBP2 (*C*) along with FLAG-PPARα. The CtBP2/PPARα complex formation was analyzed by FLAG co-immunoprecipitation. The densitometric quantification is shown to the *right* of each blot. *D* and *E*, structural modeling of acyl-CoAs/CtBP2 dimer interactions (*D*, palmitoyl-CoA; *E*, malonyl-CoA). The two molecules of the CtBP2 dimer are coded in different colors (*green* and *blue*), and the *white open oval* indicates the CoA moiety. *F*, the effects of different length of acyl chains. Acyl-CoAs (500 μM) with different length of acyl-chain (acetyl-CoA: C2, malonyl-CoA: C3, oleoyl-CoA: C18, and octanoyl-CoA: C8) were added to the cell lysates from HEK293 cells expressing WT CtBP2 along with FLAG-FoxO1. The CtBP2/FoxO1 complex formation was analyzed by FLAG-co-immunoprecipitation. CtBP, C-terminal binding protein; PPARα, peroxisome proliferator-activated receptor alpha.

We next investigated the metabolite-dependent monomer-dimer equilibrium of CtBP2. Previous studies have shown that CtBP2 adopts a dimeric conformation when bound with NADH/NAD^+^ (17) that can be decomposed into a monomeric conformation upon binding to acyl-CoAs (15). Firstly, we examined the effects of acyl-CoA–mediated monomerization of CtBP2. Since long-chain fatty acyl-CoAs can be incorporated into PPARα and modulate its activity, we tested malonyl-CoA, which has been reported to have negligible effects on PPARα activity (12) and a suppressive effect on fatty acid oxidation through the inhibition of CPT1 (13). In our preceding study, we showed that CtBP2 adopts the monomeric configuration with long-chain fatty acyl-CoAs as well as acetyl-CoA resulting in dissociation from FoxO1 (15). In agreement with this, addition of malonyl-CoA to cell lysates expressing CtBP2 and FoxO1 decreased CtBP2/FoxO1 complex formation, indicating that the conformational equilibrium was shifted toward monomer by malonyl-CoA (Fig. 2*A*). In contrast, addition of malonyl-CoA to cell lysates expressing CtBP2 and PPARα promoted the interaction, suggesting that monomeric CtBP2 preferentially binds to PPARα (Fig. 2*B*). The CtBP2 mutant lacking the Rossmann fold pocket (G189,192A) did not respond to malonyl-CoA supplementation (Fig. 2*C*), consistent with our hypothesis that malonyl-CoA modulates CtBP2 activity through binding to its Rossmann fold pocket. To further support these findings, we performed in silico structural modeling of these interactions (Fig. 2, *D* and *E*; Supporting informations 2 and 3). In this analysis, palmitoyl-CoA was found to bind to CtBP2 with its CoA moiety in the Rossmann fold and the acyl-chain moiety at the dimerization interface as reported previously (Fig. 2*D* and Supporting information 2). Indeed, the CoA moiety of malonyl-CoA was similarly accommodated in the Rossmann fold while the short acyl-chain protruded to the dimerization interface, structurally resembling acetyl-CoA that was investigated in our preceding study (15) (Fig. 2*E* and Supporting information 3). Based on our previous report (15) that accommodation of acetyl-CoA modestly shifts the conformational equilibrium of CtBP2 to monomer (15), these structural modeling further support our idea that CtBP2 adopts a monomeric conformation with malonyl-CoA. We further explored the effects of different length of acyl chains on the CtBP2 monomer-dimer equilibrium by taking advantage of the CtBP2–FoxO1 complex that was extensively investigated in our preceding study (15). The eight-carbon fatty acyl-CoA, octanoyl-CoA (C8), was as effective as the long-chain fatty acyl-CoA, oleoyl-CoA (C18) to induce the CtBP2 monomeric configuration, while the effects of two-carbon and three-carbon acyl-CoAs, acetyl-CoA (C2) and malonyl-CoA (C3) were modest (Fig. 2*F*). Despite the difficulty in the fair assessment of the effects of long-chain fatty acyl-CoAs on the CtBP2–PPARα interaction, long-chain acyl-CoAs may also increase the CtBP2–PPARα complex formation to inhibit the activity of PPARα. In addition, we examined the effect of malonyl-CoA on the interaction between CtBP2 and SREBP1. While we reported long-chain fatty acyl-CoAs suppress the CtBP2–SREBP1 interaction (15), the effect of malonyl-CoA was marginal, which may reflect the indirect nature of this interaction (15) and requirement of long acyl chains for complete monomerization of CtBP2 (Fig. S2).

**Figure 3:**
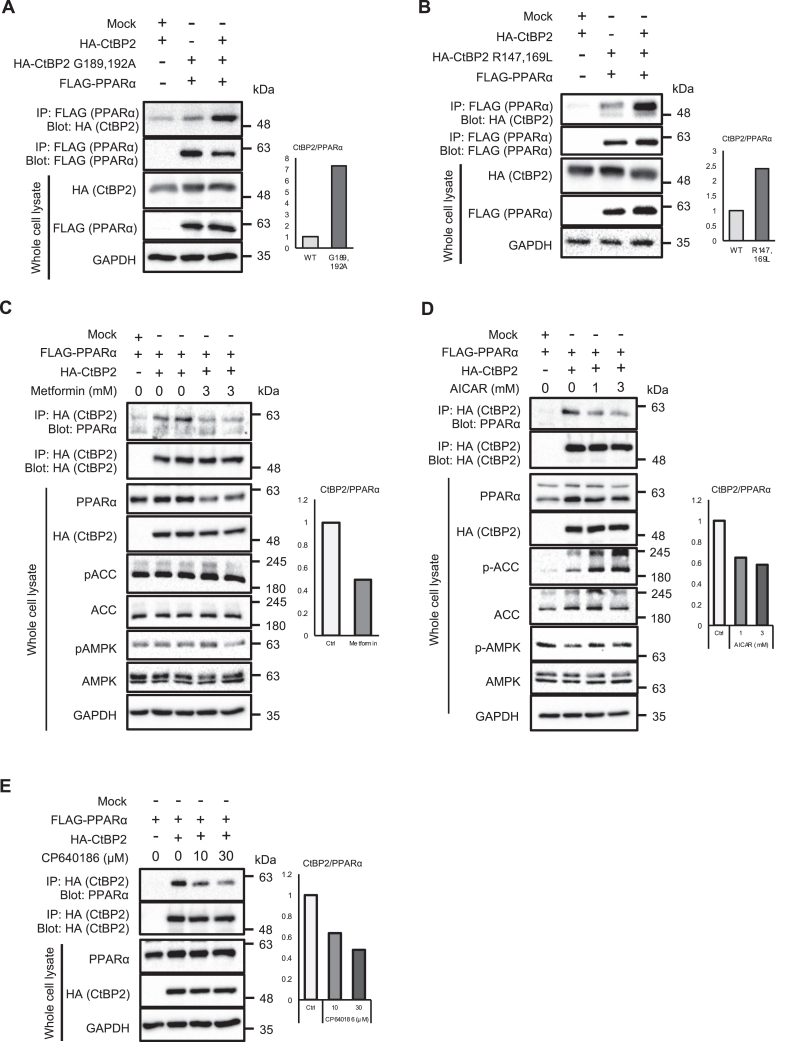
**Monomeric CtBP2 preferentially interacts with PPARα.***A*, either WT CtBP2 or Rossmann fold mutant CtBP2 (G189A, G192A) was transfected into HEK293 cells along with FLAG-PPARα. *B*, either WT CtBP2 or dimerization-defective mutant CtBP2 (R147L, R169L) was transfected into HEK293 cells along with FLAG-PPARα. *C*–*E*, WT CtBP2 and FLAG-PPARα were transfected into HepG2 cells and treated with the indicated concentrations of metformin (*C*), AICAR, an AMPK activator (*D*), or CP640186, an ACC inhibitor (*E*) for 24 h, 2 h, or 8 h. Thereafter, CtBP2/PPARα transcriptional complex was co-immunoprecipitated. The densitometric quantification is shown to the *right* of each blot. Ctrl: vehicle control (0 mM or 0 μM) of each compound. ACC, acetyl-CoA carboxylase; AICAR, 5-aminoimidazole-4-carboxamide-1-β-d-ribofuranoside; AMPK, AMP-activated protein kinase; CtBP, C-terminal binding protein; PPARα, peroxisome proliferator-activated receptor alpha.

We further took advantage of CtBP2 mutants that favor the monomeric configuration. It has been shown that mutations of the Rossmann fold (G189,192A) shifts the conformational equilibrium to monomer and that mutations at the dimeric interface (R147,169L) abrogate the dimerization (26). Both mutations robustly increased the CtBP2–PPARα interaction (Fig. 3, *A* and *B*), further supporting our proposed model. From a therapeutic point of view, mitigation of malonyl-CoA production may liberate PPARα from the repressive complex. In order to address this possibility, we stimulated HepG2 cells with metformin, an antidiabetic drug that activates AMPK to inhibit ACC, the rate-limiting enzyme of malonyl-CoA synthesis. Indeed, metformin activated this pathway, resulting in dissociation of the CtBP2–PPARα complex, suggesting the therapeutic potential of targeting this transcriptional system (Fig. 3*C*). Since both AMPK-dependent and AMPK-independent mechanisms have been reported to underlie metformin’s metabolic benefits (27), we further validated this finding with small molecules targeting this pathway. 5-aminoimidazole-4-carboxamide-1-β-d-ribofuranoside (AICAR), the most widely used activator of AMPK, decreased the CtBP2–PPARα complex formation in a dose-dependent manner (Fig. 3*D*). We also directly inactivated ACC with CP640186, a pharmacological ACC inhibitor, to decrease malonyl-CoA production. This resulted in a dose-dependent decrease of CtBP2–PPARα complex formation (Fig. 3*E*). Furthermore, 2-deoxyglucose, a competitive inhibitor of glycolysis that also activates AMPK, decreased CtBP2–PPARα complex formation (Fig. S3).

**Figure 4:**
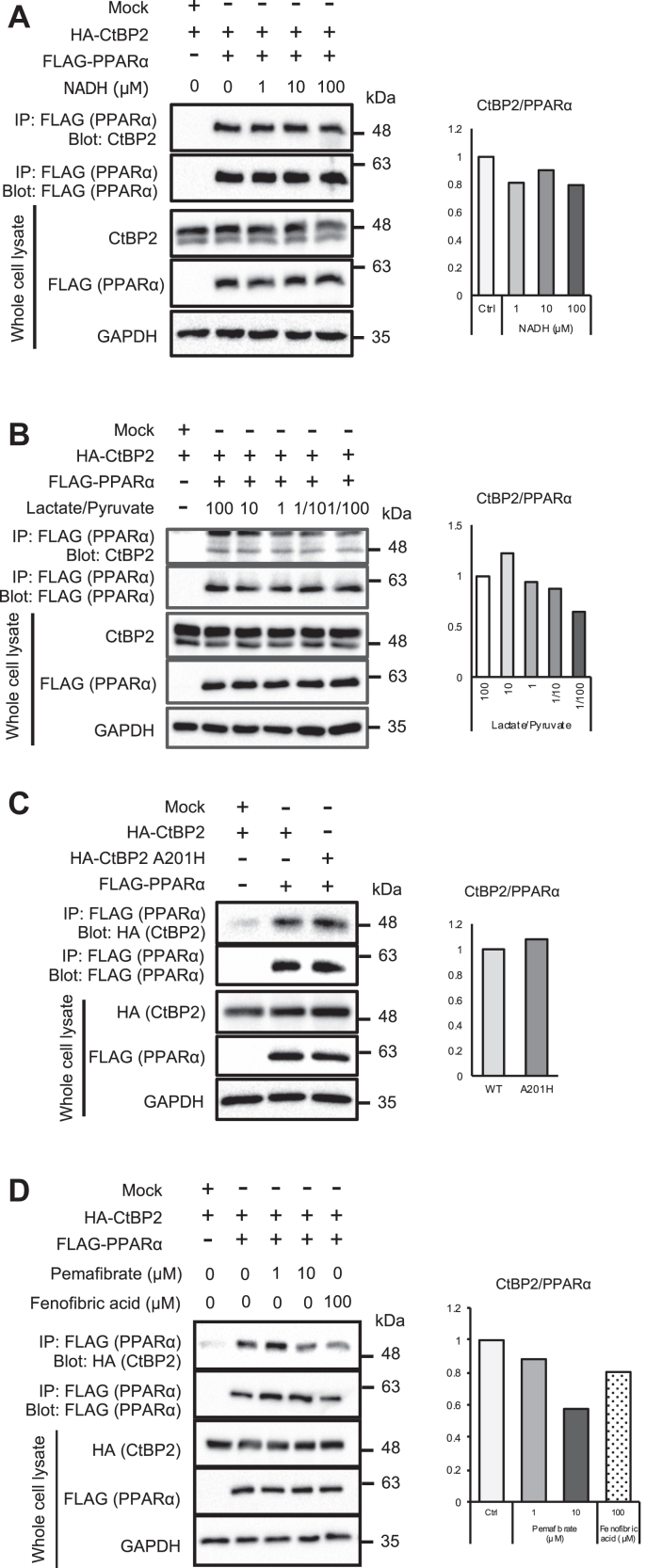
**The effects of NADH and modulation of PPARα activities on CtBP2/PPARα complex formation.***A*, increasing concentrations of NADH were added to the cell lysates from HEK293 cells expressing WT CtBP2 and FLAG-PPARα. *B*, HEK293 cells expressing WT CtBP2 and FLAG-PPARα were stimulated with different ratios of lactate/pyruvate for 1 h. *C*, either WT CtBP2 or dimerization-prone mutant CtBP2 (A201H) was transfected into HEK293 cells along with FLAG-PPARα. The CtBP2/PPARα complex formation was analyzed by FLAG co-immunoprecipitation (*A*–*C*). *D*, WT CtBP2 and FLAG-PPARα were transfected into HEK293 cells and treated with the indicated concentrations of pemafibrate or fenofibrate to activate PPARα for 24 h. Thereafter, CtBP2/PPARα transcriptional complex was co-immunoprecipitated. The densitometric quantification is shown to the *right* of each blot. The densitometric quantification is shown to the *right* of each blot. CtBP, C-terminal binding protein; PPARα, peroxisome proliferator-activated receptor alpha.

Since CtBP2 has been reported to respond to the ratio of NADH/NAD^+^, we also examined the possible involvement of CtBP2’s pyridine dinucleotide-sensing property (19). The effect of NADH supplementation in cell lysates was relatively marginal (Fig. 4*A*). We further modulated the NADH/NAD^+^ ratio in live cells by changing the extracellular lactate/pyruvate ratio, based on the fact that lactate dehydrogenase is an equilibrium enzyme coupling the conversion of pyruvate to lactate with NADH to NAD^+^ (28). Again, an increase in the NADH/NAD^+^ ratio induced by an increase of the extracellular lactate/pyruvate ratio had a negligible effect on CtBP2–PPARα complex formation (Fig. 4*B*). We also tested A201H mutant of CtBP2 that favors the dimeric configuration (15) and found that the A201H CtBP2 was comparable to WT CtBP2 (Fig. 4*C*). Collectively, NADH/NAD^+^ supplementation had little, if any, effect on the CtBP2–PPARα interaction in these experimental settings.

Lastly, we examined the effects of PPARα activation on the CtBP2–PPARα interaction. The activation of PPARα with PPARα agonist fibrates reduced the CtBP2–PPARα complex formation (Fig. 4*D*).

### The CtBP2–PPARα complex is increased in the liver of obese mice

**Figure 5:**
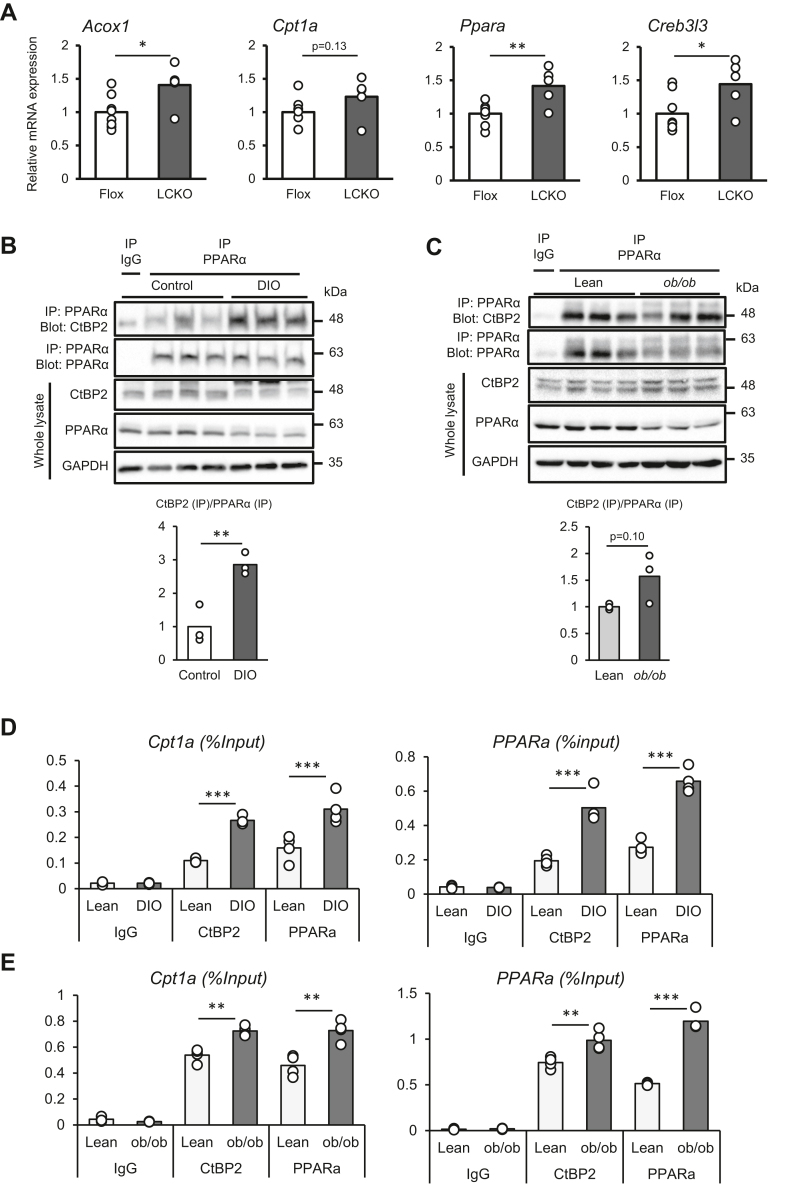
**The *in vivo*relevance of the CtBP2/PPARα interaction.***A*, the expression levels of PPARα target genes in liver-specific CtBP2 KO mice (LCKO) (n = 8 and n = 5 for flox and LCKO, respectively). Liver samples were collected after 5 to 6 h of food withdrawal. *B* and *C*, liver homogenates from high fat diet-induced obese mice (DIO, *B*), and genetically obese mice (*ob/ob*, *C*) were subjected to co-immunoprecipitation to analyze the endogenous CtBP2/PPARα complex. The densitometric quantification is shown to the *right* of each blot. *D* and *E*, recruitment of CtBP2 and PPARα to *Cpt1* and *Ppara* gene promoters analyzed by ChIP. Chromatin was obtained from liver tissues of DIO (*D*) and *ob/ob* mice (*E*) along with their lean controls. Data are expressed as the mean ± SEM. ∗, ∗∗, and ∗∗∗ denotes *p* < 0.05, *p* < 0.01, and *p* < 0.001 evaluated by unpaired two-tailed Student’s *t* test. ChIP, chromatin immunoprecipitation; CPT, carnitine palmitoyltransferase; CtBP, C-terminal binding protein; PPARα, peroxisome proliferator-activated receptor alpha.

Having observed these *in vitro* findings, we investigated the *in vivo* relevance of this transcriptional complex. As a first attempt, we examined gene expression in the liver-specific CtBP2 KO mice (15). Indeed, genetic deletion of CtBP2 in the liver increased the expression of PPARα target genes (1.2 ∼ 1.4-fold increase), reflecting the liberation of PPARα from CtBP2-mediated repression, although the difference did not reach statistical significance for *Cpt1a* gene (*p* = 0.13) (Figs. 5*A* and S4).

**Figure 6:**
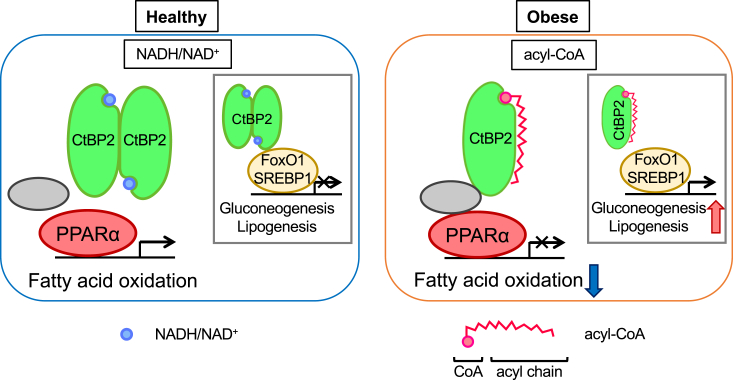
**Schematic representation of our proposed model.** Our previous study showed that monomeric CtBP2, the predominant form in obesity, dissociates from FoxO1 and SREBP1, resulting in increased expression of the gluconeogenic and lipogenic programs. In this study, we demonstrate that monomeric CtBP2 interacts with PPARα to repress its activity and illustrate a critical role of malonyl-CoA in this context. *Gray circle* indicates the unidentified intermediary molecule(s) between CtBP2 and PPARα. CtBP, C-terminal binding protein; PPARα, peroxisome proliferator-activated receptor alpha; SREBP, sterol regulatory element-binding protein.

We next examined CtBP2–PPARα complex formation in the livers of multiple animal models of obesity. In the liver of high fat diet-induced obese mice, the protein expression of PPARα was decreased, potentially reflecting reduced PPARα activity. In accordance with our hypothesis, the CtBP2–PPARα interaction was increased in the livers of obese mice (2.8-fold increase based on our densitometric quantification, Fig. 5*B*). Similarly, in the livers of genetically obese mice, the protein expression levels of PPARα were reduced. Even in the presence of this reduced protein expression, CtBP2 binding to PPARα was maintained. In other words, CtBP2 bound to PPARα on a per molecule basis tended to be increased in mice with genetic obesity (1.6-fold increase based on our densitometric quantification, *p* = 0.10, Fig. 5*C*). To further clarify the interplay between CtBP2 and PPARα in the promoters, we performed chromatin immunoprecipitation (ChIP) experiments. Despite the decreased protein expression, the recruitment of PPARα to the promoters of its target genes was increased in the liver of both diet-induced obese and *ob/ob* mice (Fig. 5, *D* and *E*). Importantly, CtBP2 recruitment to those promoters was also increased in obesity (Fig. 5, *D* and *E*). These data further support our hypothesis that CtBP2 is recruited to those promoters to repress PPARα in obesity. As demonstrated in our previous finding that CtBP2 adopts a monomeric state in obese liver due to acyl-CoA deposition (15), the findings in this study further indicate that CtBP2 represses the transcriptional activity of PPARα particularly in the liver of obesity (Fig. 6).

## Discussion

In this study, we identified an interaction between CtBP2 and PPARα that is increased in obese liver to repress PPARα transcriptional activity. Our findings demonstrated a sequential event whereby CtBP2 adopts a monomeric configuration in response to obesity-induced metabolic alterations, resulting in binding to PPARα. Through this interaction, CtBP2 represses PPARα, which may contribute to hepatic steatosis and other metabolic inflexibilities in obese liver (Fig. 6) (29).

The critical roles of malonyl-CoA in energy metabolism have been reported with a particular emphasis on the inhibition of CPT1 (13). Our findings indicate that malonyl-CoA governs fatty acid oxidation by multiple systems. In addition to malonyl-CoA, long-chain fatty acyl-CoAs may have inhibitory roles in fatty acid oxidation through CtBP2, although there were technical challenges since both CtBP2 and PPARα can accommodate fatty acyl-CoAs in their structural cavities. Despite this technical difficulty, fatty acyl-CoA–mediated suppression of PPARα activity appears to be plausible, according to a previous report (12). It is also of note that CtBP2 provides an additional lipid-binding cavity to PPARα. There has been debate as to whether PPARα responds to endogenous lipids derived from *de novo* lipogenesis (30, 31) or exogenously supplied lipids. Our findings may offer some clues to resolve this debate.

CtBP2 may confer pyridine nucleotide-sensing capability to PPARα, although we were not able to observe this possibility in this study. Cytosolic NADH production is tightly coupled with glycolysis, which may be saturated under regular cell culture conditions with high glucose in highly glycolytic tumor-derived cell lines. The indirect nature of the interaction between CtBP2 and PPARα may have some influence on this. We also need to acknowledge some residual controversy surrounding the capability of CtBP2 to discriminate between NADH and NAD^+^ (32, 33, 34). To the best of our knowledge, the molecular link between PPARα and pyridine nucleotide metabolism has not been reported, therefore deserves further investigation.

It is known that the expression of PPARα is driven by a positive autoregulatory system (35). While this feedback loop serves as a self-amplifying system, CtBP2 may confer a self-extinguishing capability to PPARα in this context. Suppression of PPARα activity would decrease fatty acid oxidation (36, 37) as well as malonyl-CoA catabolism (38, 39), leading to the accumulation of fatty acyl-CoAs and malonyl-CoA. These metabolic alterations would increase the CtBP2–PPARα interaction through the conformational equilibrium shift of CtBP2 toward monomers, leading to further suppression of PPARα activity. The lipid spillover into the liver in obesity may trigger this autoloop system, which may at least in part contribute to metabolic deterioration.

CtBP2 functions in a dynamic equilibrium between dimer and monomer, and in most cases, the repressor activity is potentiated upon dimerization (18, 40) with some exceptions as observed in this case. CtBP2 adopts a monomeric configuration in obesity and dissociates from FoxO1 and SREBP1 (15) (Fig. 6). In this context, the genetic deletion of CtBP2 may mimic obesity-induced conformational alterations of CtBP2. However, it may not faithfully recapitulate obesity since monomeric CtBP2 is rather a gain-of-function state in the interaction with PPARα. Thus, we have to be prudent in evaluating the roles of CtBP2 using the genetic deletion model.

One of the issues that remain to be solved is the intermediary molecule(s) between CtBP2 and PPARα. We were not able to find the putative CtBP-binding motif (23) in the amino acid sequence of retinoid X receptor, an obligatory heterodimerization partner of PPARα (41). It was reported that nuclear receptor corepressor (NCOR) is recruited to PPARα when forming a repressor complex (37), and we indeed found the CtBP-binding motif in NCOR sequences. Therefore, NCOR may be a prime candidate intermediary molecule. Since the possible intermediary molecule(s) may confer redundancies and complexities to the CtBP2-mediated PPARα repression, this issue deserves further scrutiny.

One of the most established biological roles of PPARα is fatty acid–activated transcriptional regulation that supports fatty acid oxidation and ketogenesis in response to fasting (36, 37). One of the fasting-induced metabolic signatures, reduced ATP/AMP ratio, activates AMPK, which in turn also enhances PPARα transcriptional activity (42, 43, 44). In contrast to this physiological regulation of PPARα, there have been several proposed models for the reduced PPARα activity observed in obesity, albeit with some controversy (45, 46, 47). Elevated S6 kinase 2 activity in obese liver suppresses PPARα through recruiting NCOR (48). The reduced hepatic adiponectin signaling that activates the AMPK–PPARα pathway may also explain the attenuation of PPARα activity in obesity (49). Despite the existence of these and other models that may also contribute to this pathogenesis (31), there may exist more redundancies, including the CtBP2/PPARα system proposed in this study. We reported the therapeutic potential of CtBP2 dimerization in obese liver to ameliorate diabetes as well as steatosis (15). Thus, small molecule–mediated CtBP2 dimerization may provide attractive metabolic benefits such as increased fatty acid oxidation.

In conclusion, we identified a novel interaction between CtBP2 and PPARα that responds to metabolic alterations induced by obesity. Our findings in this study provide a new conceptual framework to understand the pathogenesis of obesity that can be exploited to develop therapeutic approaches.

## Experimental procedures

### Plasmids and cells

Human PPARα and CtBP2 complementary DNAs (cDNAs) were amplified by PCR with N-terminal FLAG-tag and N-terminal hemagglutinin (HA) tag, respectively, and cloned into pcDNA3.1 (+) (Thermo Fisher Scientific, V79020). The following CtBP2 mutants were generated using the Q5 Site-Directed Mutagenesis Kit (New England Biolabs, E0554S): Rossmann fold-defective mutant (Gly189Ala and Gly192Ala), dimerization-defective mutant (Arg147Leu and Arg169Leu) (26), and dimerization-prone mutant (Ala201His) (15).

HEK293 human embryonic kidney cells and HepG2 human hepatoma cells were cultured in Dulbecco's modified Eagle's medium (Gibco, 11965) containing 25 mM glucose, 100 U/ml penicillin, and 100 μg/ml streptomycin sulfate supplemented with 10% fetal bovine serum.

HEK293 cells (2 × 10^5^ cells/ml) were plated into each well of a 6-well plate and cultured for 24 h. The cells were then transiently transfected with a control plasmid, FLAG WT PPARα (50) along with either WT CtBP2 or mutated CtBP2 using lipofectamine LTX (Thermo Fisher Scientific, 15338) for 48 h. To examine the effects of extracellular lactate/pyruvate ratios, cells were cultured with the indicated ratios of lactate/pyruvate for 1 h.

HepG2 cells (1 × 10^5^ cells/ml) were plated into each well of a 24-well plate and cultured for 24 h. The cells were then transduced with Ad-beta-GUS or Ad-human CtBP2-HA (1 × 10^9^ VP/ml) for 48 h. Thereafter, cells were treated with either vehicle or 10 μM pemafibrate (Kowa Co Ltd) for 24 h.

HepG2 cells (2 × 10^5^ cells/ml) were plated into each well of a 12-well plate and cultured for 24 h. The cells were then transiently transfected with a control plasmid, FLAG WT PPARα along with WT CtBP2 using lipofectamine 3000 (Thermo Fisher Scientific, L3000008) for 48 h. To examine the effect of AICAR (1 mM and 3 mM, Wako 015-22531), CP640186 hydrochloride (10 μM and 30 μM, Medchemexpress HY-15259A), and metformin hydrochloride (3 mM, TCI M2009), cells were treated with the indicated concentrations of these reagents for 2 h, 8 h, and 24 h, respectively. Cells were treated with 10 mM 2-deoxyglucose-D-glucose (Nacalai, 10722M) to alternatively activate AMPK. Thereafter, the cell lysates were subjected to co-immunoprecipitation.

### Animals

The research protocol was approved by the Animal Care Committee, University of Tsukuba, and all experimental procedures involving animals were conducted according to the guidelines. All mice used were male and maintained on a 14-h light and 10-h dark period cycle. Leptin-deficient *ob/ob* mice (B6. Cg-Lep ob/J, 10 weeks of age upon euthanasia) were purchased from the Jackson Laboratories (Stock #000632). Mice were fed a high-fat diet (D12492, Research Diets) for 12 weeks starting from 4 weeks of age. Liver-specific CtBP2-deficient mice (LCKO, 8 weeks of age) were generated as described previously (15).

### Western blot analysis and co-immunoprecipitation experiments

Proteins were extracted from cells or liver samples with buffer A (50 mM Tris–HCl pH 7.4, 150 mM NaCl, 1% Nonidet P-40, 1 mM EDTA, 10 mM NaF, 2 mM Na_3_VO_4_) with protease inhibitor cocktail (Sigma-Aldrich, P8340) and subjected to SDS-PAGE. Membranes were incubated with the following antibodies: anti-CtBP2 (BD, 612044), anti-PPARα (Santa Cruz, sc-398394), anti-GAPDH (Santa Cruz, sc-32233), anti-SREBP1 (Novus, NB600-582), anti-FLAG (Sigma-Aldrich, F3165), and anti-HA (Cell Signaling, 3724S).

The membranes were incubated with secondary antibody conjugated with horseradish peroxidase (Cell Signaling, 7074S and 7076S) and were visualized using ChemiDoc XRS Plus System (Bio-Rad). To detect endogenous binding of PPARα and CtBP2, PPAR alpha antibody (GeneTex, GTX101098) or Rabbit IgG isotype control (GeneTex, GTX35035) were cross-linked to Dynabeads Protein G (Invitrogen, 10004D) with 50 mM dimethyl pimelimidate (Sigma-Aldrich, D8388). Liver samples were lysed with buffer A, and the protein complexes were immunoprecipitated in buffer A with a reduced concentration of NP40 (0.5%) (50 mM Tris–HCl pH 7.4, 150 mM NaCl, 0.5% Nonidet P-40, 1 mM EDTA, 10 mM NaF, 2 mM Na_3_VO_4_) for 2 h at 4 °C. The beads were washed four times with buffer A containing 0.5% NP40, eluted with SDS loading buffer, and analyzed by Western blot analysis.

HEK293 cells were transiently transfected with either a control plasmid or FLAG WT PPARα along with either HA WT CtBP2, HA mutant CtBP2 using lipofectamine LTX (Thermo Fisher Scientific, 15338).

Cells expressing the indicated plasmids were lysed with buffer A containing 1% NP40 and immunoprecipitated with FLAG M2 magnetic beads (MBL, M185-11R) or anti-HA magnetic beads (Thermo Fisher Scientific, 88836) in buffer A with 0.5% NP40 for 2 h at 4 °C. The beads were washed four times with buffer A containing 0.5% NP40 and eluted with 0.5 mg/ml of 3x FLAG peptide (Sigma, F4799) or HA peptide (MBL, 3320-205). To evaluate the effects of malonyl-CoA (Sigma, M4263) and NADH (Sigma, N8129), the cell lysates were immunoprecipitated with FLAG M2 magnetic beads with increasing concentrations of malonyl-CoA or NADH for 4 h or 8 h at 4 °C. Thereafter, the PPARα–CtBP2 complex was eluted and analyzed.

### Quantitative real-time PCR

Total RNA was isolated using Sepasol-RNA I Super G (Nacalai, 09379), and cDNA was synthesized with PrimeScript RT Master Mix (Takara Bio, RR036A). Quantitative real-time PCR analysis was performed using SYBR Green in a Thermal Cycler Dice Real-Time System (Takara Bio, RR820A). Data were normalized to peptidylprolyl isomerase A (*Cyclophilin A*) or ribosomal protein, large, P0 (*36B4*) expression. The primer sequences were as follows.

**Table:** List of primers for our quantitative PCR analysis

*Gene*		Sequence
Rplp0	Forward	5′-GTCACTGTGCCAGCTCAGAA-3′
	Reverse	5′-CTCCCACCTTGTCTCCAGTC-3′
Cpt1a	Forward	5′-TTGGAAGTCTCCCTCCTTCA-3′
	Reverse	5′-GCCCATGTTGTACAGCTTCC-3′
Acox1	Forward	5′-CGATCCAGACTTCCAACATGAG-3′
	Reverse	5′-CCATGGTGGCACTCTTCTTAACA-3′
Ppara	Forward	5′-ACGCGAGTTCCTTAAGAACCTG-3′
	Reverse	5′-GTGTCATCTGGATGGTTGCTCT-3′
Creb3l3	Forward	5′-CCTGTTTGTCGGCAGGAC-3′
	Reverse	5′-CGGGGGACGATAATGGAGA-3′
Abca1	Forward	5′-AAAACCGCAGAGACATCCTTCAG-3′
	Reverse	5′-CATACCGAAACTCGTTCACCC-3′
Apoa1	Forward	5′-TCACCCACACCCTTCAC-3′
	Reverse	5′-CTGGCTCCCTGTCAGGAAGA-3′
Apoa5	Forward	5′-GCGAGTTCTGCCGTAG-3′
	Reverse	5′-CCCAACCCCATCAAATGTGA-3′
Apoc2	Forward	5′-CCAAGGAGGTTGCCAAAGAC-3′
	Reverse	5′-TGCCTGCGTAAGTGCTCATC-3′
Anglpt4	Forward	5′-CATCCTGGGACGAGATGACT-3′
	Reverse	5′-TGACAAGCGTTACCACAGGC-3′
Cyp7a1	Forward	5′-GCTGAGAGCTTGAAGCACAAGA-3′
	Reverse	5′-TTGAGATGCCCAGAGGATCAC-3′
Fgf21	Forward	5′-CCTCTAGGTTTCTTTGCCAAC-3′
	Reverse	5′-AAGCTGCAGGCCTCAAGG-3′
Vnn1	Forward	5′-CACCGGGGTAGAGCCAAATCT-3′
	Reverse	5′-GATCGTATCTGCAGCGAAGC-3′
PPIA	Forward	5′-AGTCCATCTATGGGGAGAAATTTG-3′
	Reverse	5′-GCCTCCACAATATTCATGCCTTC-3′
CPT1A	Forward	5′-ACAACAAAAGCCCCTGACTG-3′
	Reverse	5′-AGGGCAGAGAGAGCTACATCC-3′
ACOX1	Forward	5′-CCCAGACAGAGATGGGTCAT-3′
	Reverse	5′-TCCTGGGTTTCAGGGTCATA-3′
CTBP2	Forward	5′-ACACCATCACCCTCACCAG-3′
	Reverse	5′-TGTTGCACACGGCAATTC-3′
PPARA	Forward	5′-TGGACCTGAACGATCAAGTGA-3′
	Reverse	5′-CCCATTTCCATACGCTACCAG-3′
CREB3L3	Forward	5′-CCTCTGTGACCATAGACCTGG-3′
	Reverse	5′-ACGGTGAGATTGCATCGTGG-3′

### PPRE luciferase reporter assay

HEK293 cells (5 × 10^4^ cells/ml) were plated into each well of a 48-well plate and cultured for 24 h. The cells were then cotransfected with 50 ng of a PPRE luciferase reporter plasmid and 5 ng of a pRL-SV40 plasmid encoding *Renilla* (Promega, E2231) using Lipofectamine LTX with Plus Reagent. For overexpression, cells were cotransfected with 100 ng of a control plasmid or WT CtBP2 along with 100 ng of a control plasmid, WT PPARα, PPARγ, or PPARδ. Cells were incubated for 48 h after the transfection, and luciferase activities were measured in a Synergy HTX Multi-Mode Reader (BioTek), using the Dual-Luciferase Reporter Assay System (Promega, E1960). The PPRE luciferase activities were normalized to *Renilla* activities.

### Structural prediction of CtBP2 with acyl-CoAs by docking simulation

The X-ray structure of CtBP2 dimer (PDB ID: 4LCJ) was downloaded from the Protein Data Bank (PDB). Assignment of bond orders and hydrogenation, hydrogen bond optimization, and energy minimization were performed by Protein Preparation Wizard in Maestro (Schrödinger, LLC) as described previously (15). CtBP2–malonyl-CoA complex structure was created by docking simulation using Glide (51). Prepared 4LCJ A-chains were used for docking simulations. The grid box center coordinates with each side of 20 Å ware set to 17.28, −3.85, 7.8. Positional constraints were set on the adenine ring and phosphorus atom of NADH bound to the A chain to output a docking pose where the adenine ring and phosphate of malonyl-CoA overlap. The CtBP2/malonyl-CoA and CtBP2/palmitoyl-CoA monomeric models were aligned on the A and B chains of the CtBP2 X-ray structure (PDB code: 4LCJ). Energy minimization calculation was performed on the two aligned structures, and energy minimized structures were used as the CtBP2/malonyl-CoA and CtBP2/palmitoyl-CoA dimeric forms.

### Chromatin immunoprecipitation

Liver chromatin was obtained from liver tissues as reported previously (15). ChIP assay was carried out using Magna ChIP HiSens Chromatin Immunoprecipitation system (EMD Millipore) with minor modifications (15). Chromatin was immunoprecipitated either with control IgG (Cell Signaling), anti-CtBP2 (Active Motif, 61261), and anti-PPARα (Abcam, ab227074). Immunoprecipitated DNA and input DNA were quantified by real-time PCR with primers specific for *Cpt1a* or *Ppara* gene promoters (primer sequences are as follows: *Cpt1a* forward: 5′- gggtccctgcagtatagcct -3′, *Cpt1a* reverse: 5′- acccacctgcccttgaac -3′, *Ppara* forward: 5′- tgcgatctagaccagctcaac 3′, *Ppara* reverse: 5′- ggccaggactgaagttcaag -3′).

### Measurement of oxygen consumption

HepG2 cells (5 × 10^5^ cells/ml) were plated into each well of a 96-well black bottom plate and cultured for 24 h. The cells were then transduced with Ad-GUS or Ad-human CtBP2-HA (1 × 10^9^ VP/ml) for 48 h. OCR was measured according to the instruction manual of the Extracellular OCR Plate Assay Kit (Dojindo E297). The cells were treated with either control bovine serum albumin or bovine serum albumin-conjugated palmitate (200 μM) for 30 min, and the fluorescent signals were measured at 10 min intervals. The calculation of OCR was derived from an analysis of the kinetic profiles obtained from measurements.

### Statistical analysis

Statistical differences between two groups were analyzed using Student’s *t* test. Statistical differences between more than three groups were analyzed using one-way ANOVA followed by Tukey’s multiple comparisons test. The bar graphs with error bars represent means ± SEM. Significance is indicated by asterisks: ∗*p* < 0.05, ∗∗*p* < 0.01, ∗∗∗*p* < 0.001 and sharp: #*p* < 0.05, ##*p* < 0.01, ###*p* <0.001.

## Data availability

All data contained within the manuscript are available upon reasonable request to M. S. (msekiya@md.tsukuba.ac.jp).

## Supporting information

This article contains supporting information.

## Conflict of interest

The authors declare that they have no conflict of interest with the contents of this article.

## Supporting information

Supporing information
