# Supplementary material for: Obesity-induced metabolic imbalance allosterically modulates CtBP2 to inhibit PPAR-alpha transcriptional activity
Source: J Biol Chem. 2023 Jun 5;299(7):104890. doi: 10.1016/j.jbc.2023.104890 (PMC10339064; doi:10.1016/j.jbc.2023.104890)
Supplement: Supporing information [file mmc1.docx]

**Supporting information**

**Title: Obesity-induced metabolic imbalance allosterically modulates CtBP2 to inhibit PPAR-alpha transcriptional activity.**

Authors: Kenji Saito^1^, Motohiro Sekiya^1,#^, Kenta Kainoh^1^, Ryunosuke Yoshino^2^, Akio Hayashi^1^, Song-Iee Han^1^, Masaya Araki^1^, Hiroshi Ohno^1^, Yoshinori Takeuchi^1^, Tomomi Tsuyuzaki^1^, Daichi Yamazaki^1^, Chen Wanpei^1^, Lisa Hada^1^, Sho Watanabe^1^, Putu Indah Paramita Adi Putri^1^, Yuki Murayama^1^, Yoko Sugano^1^, Yoshinori Osaki^1^, Hitoshi Iwasaki^1^, Naoya Yahagi^1^, Hiroaki Suzuki^1^, Takafumi Miyamoto^1^, Takashi Matsuzaka^1,2^, Hitoshi Shimano^1^

**Supporting information 1**

**Supplementary Figures.**

**Supplementary Figure S1. CtBP2 represses PPARα transcriptional activities.**

**A**. The responses of the PPRE luciferase reporter to all three PPAR isoforms in the presence or absence of CtBP2 expression in HEK293 cells (n=4). **B**. HepG2 hepatoma cells were transduced with adenoviruses expressing control protein GUS or CtBP2 in the presence or absence of the indicated concentrations of GW7647 or pemafibrate (n = 5 for each group). The expression levels of key genes were analyzed. Data are expressed as the mean ± SEM. *, ** and *** denote p < 0.05, p < 0.01, and p < 0.001 evaluated by one-way ANOVA followed by Tukey's multiple comparisons test. ns denotes non-statistical significance.

**Supplementary Figure S2. The effect of malonyl-CoA on CtBP2/SREBP1 complex formation.**

Indicated concentrations of malonyl-CoA or oleoyl-CoA were added to HEK293 cell lysates expressing CtBP2 and nuclear form of SREBP1a, and the CtBP2/SREBP1 complex formation was analyzed by co-immunoprecipitation.

**Supplementary Figure S3. AMPK activation reduces CtBP2/PPARα complex formation.**

Wild-type CtBP2 and FLAG-PPARα were transfected into HEK293 cells and treated with the indicated concentrations of AICAR or 2-DG to activate AMPK for 2 h. Thereafter, CtBP2/PPARα transcriptional complex was co-immunoprecipitated. The densitometric quantification is shown to the right of each blot.

**Supplementary Figure S4. Gene expression profiles in the livers with CtBP2 deficiency.**

Expression levels of PPARα target genes in liver-specific CtBP2 knockout mice (LCKO) (n = 8 and n = 5 for flox and LCKO, respectively). Liver samples were collected after 5-6 h of food withdrawal. Data are expressed as the mean ± SEM. * and ** denote p < 0.05 and p < 0.01 evaluated by one-way ANOVA followed by Tukey's multiple comparisons test. ns denotes non-statistical significance.

**Supporting information 2**

The structural modeling of CtBP2 dimer accommodating palmitoyl-CoA in the PDB file format.

**Supporting information 3**

The structural modeling of CtBP2 dimer accommodating malonyl-CoA in the PDB file format.
