# Supplementary figures and images for: Obesity-induced metabolic imbalance allosterically modulates CtBP2 to inhibit PPAR-alpha transcriptional activity
Source: J Biol Chem. 2023 Jun 5;299(7):104890. doi: 10.1016/j.jbc.2023.104890 (PMC10339064; doi:10.1016/j.jbc.2023.104890)

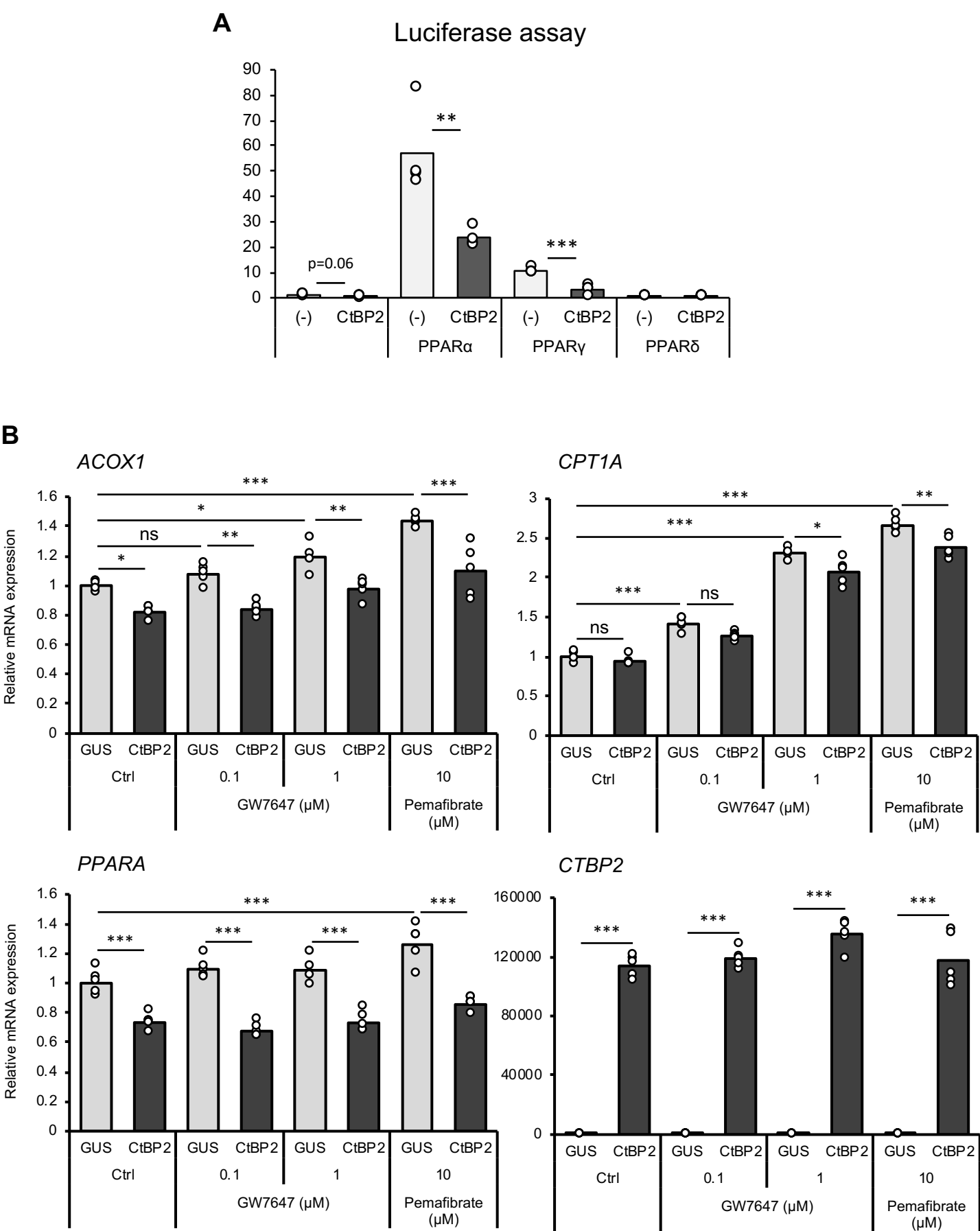

Supplementary Fig. S1

Supplement: Supporting Figure S1 [file mmc2.pdf]

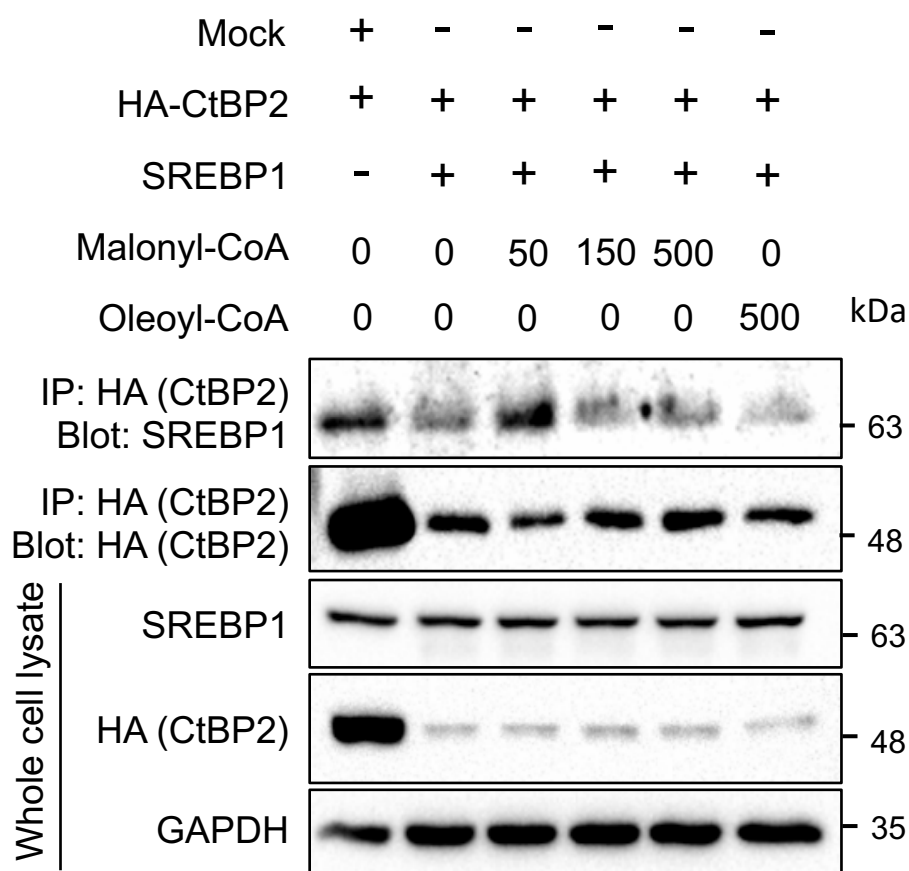

**Supplementary Fig. S2**

Supplement: Supporting Figure S2 [file mmc3.pdf]

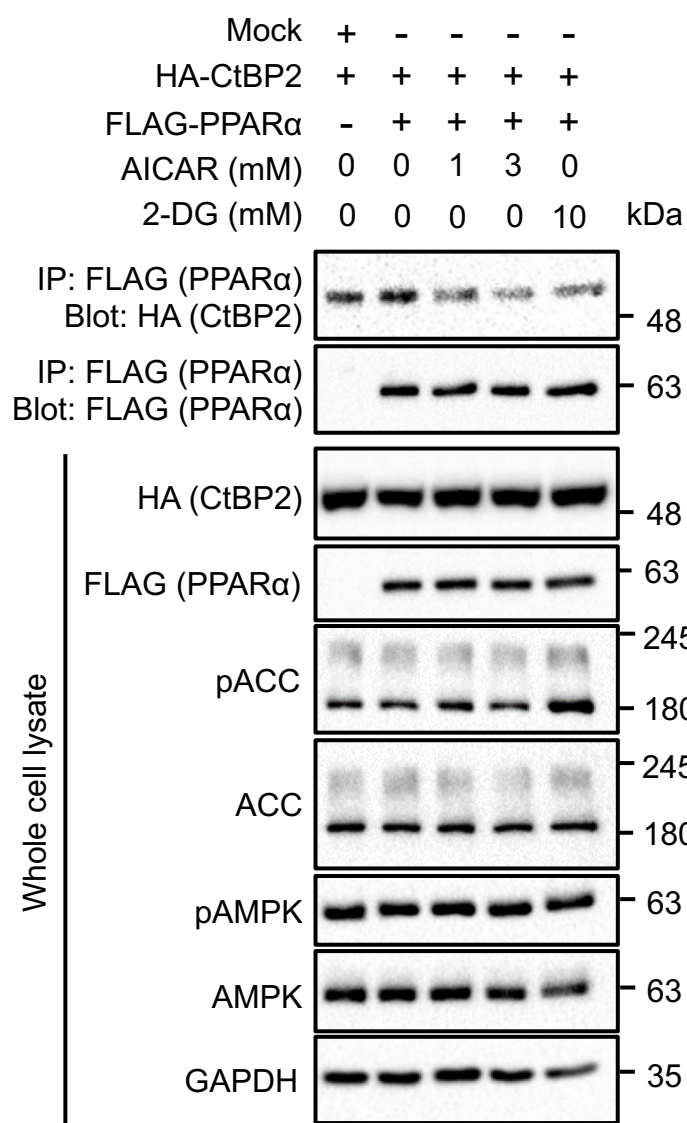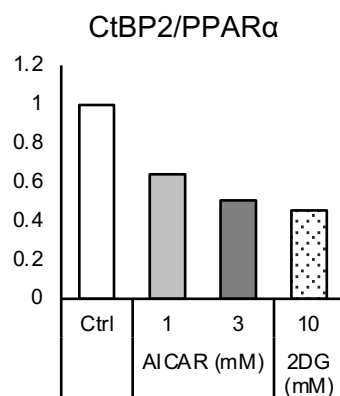

**Supplementary Fig. S3**

Supplement: Supporting Figure S3 [file mmc4.pdf]

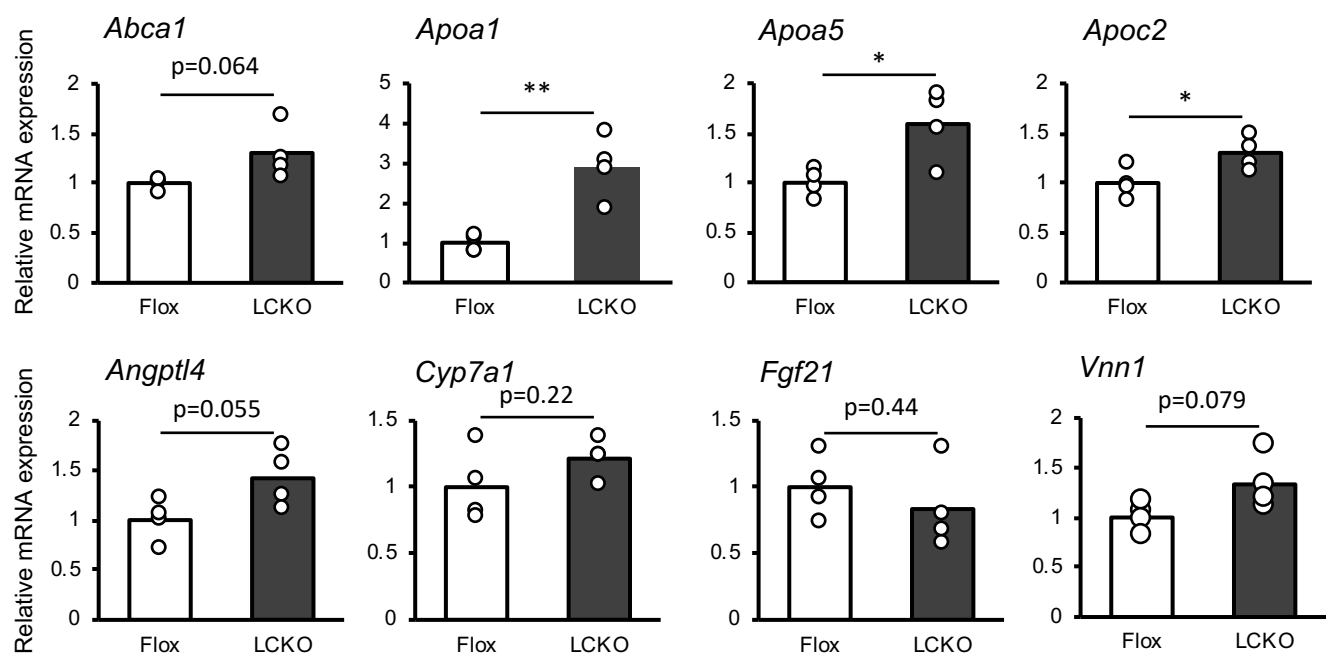

Supplement: Supporting Figure S4 [file mmc5.pdf]
